# Post-mortem tissue proteomics reveals the pathogenesis of multi-organ injuries of COVID-19

**DOI:** 10.1093/nsr/nwab143

**Published:** 2021-08-10

**Authors:** Yang Qiu, Di Wu, Wanshan Ning, Jiqian Xu, Ting Shu, Muhan Huang, Rong Chen, Jiancheng Zhang, Yang Han, Qingyu Yang, Ruiting Li, Xiaobo Yang, Yaxin Wang, Xiaojing Zou, Shangwen Pan, Chaolin Huang, Yu Xue, You Shang, Xi Zhou

**Affiliations:** State Key Laboratory of Virology, Wuhan Institute of Virology, Chinese Academy of Sciences, China; Joint Laboratory of Infectious Diseases and Health, Wuhan Institute of Virology and Wuhan Jinyintan Hospital, Wuhan Institute of Virology, Chinese Academy of Sciences, China; Center for Translational Medicine, Wuhan Jinyintan Hospital, China; State Key Laboratory of Virology, Wuhan Institute of Virology, Chinese Academy of Sciences, China; Joint Laboratory of Infectious Diseases and Health, Wuhan Institute of Virology and Wuhan Jinyintan Hospital, Wuhan Institute of Virology, Chinese Academy of Sciences, China; MOE Key Laboratory of Molecular Biophysics, Hubei Bioinformatics and Molecular Imaging Key Laboratory, Center for Artificial Intelligence Biology, College of Life Science and Technology, Huazhong University of Science and Technology, China; Department of Clinical Laboratory, Union Hospital, Tongji Medical College, Huazhong University of Science and Technology, China; Department of Critical Care Medicine, Union Hospital, Tongji Medical College, Huazhong University of Science and Technology, China; Joint Laboratory of Infectious Diseases and Health, Wuhan Institute of Virology and Wuhan Jinyintan Hospital, Wuhan Institute of Virology, Chinese Academy of Sciences, China; Center for Translational Medicine, Wuhan Jinyintan Hospital, China; State Key Laboratory of Virology, Wuhan Institute of Virology, Chinese Academy of Sciences, China; Joint Laboratory of Infectious Diseases and Health, Wuhan Institute of Virology and Wuhan Jinyintan Hospital, Wuhan Institute of Virology, Chinese Academy of Sciences, China; Center for Translational Medicine, Wuhan Jinyintan Hospital, China; Joint Laboratory of Infectious Diseases and Health, Wuhan Institute of Virology and Wuhan Jinyintan Hospital, Wuhan Jinyintan Hospital, China; Department of Critical Care Medicine, Union Hospital, Tongji Medical College, Huazhong University of Science and Technology, China; Center for Translational Medicine, Wuhan Jinyintan Hospital, China; Joint Laboratory of Infectious Diseases and Health, Wuhan Institute of Virology and Wuhan Jinyintan Hospital, Wuhan Jinyintan Hospital, China; Joint Laboratory of Infectious Diseases and Health, Wuhan Institute of Virology and Wuhan Jinyintan Hospital, Wuhan Institute of Virology, Chinese Academy of Sciences, China; Center for Translational Medicine, Wuhan Jinyintan Hospital, China; Joint Laboratory of Infectious Diseases and Health, Wuhan Institute of Virology and Wuhan Jinyintan Hospital, Wuhan Jinyintan Hospital, China; Department of Critical Care Medicine, Union Hospital, Tongji Medical College, Huazhong University of Science and Technology, China; Department of Critical Care Medicine, Union Hospital, Tongji Medical College, Huazhong University of Science and Technology, China; Department of Critical Care Medicine, Union Hospital, Tongji Medical College, Huazhong University of Science and Technology, China; Department of Critical Care Medicine, Union Hospital, Tongji Medical College, Huazhong University of Science and Technology, China; Department of Critical Care Medicine, Union Hospital, Tongji Medical College, Huazhong University of Science and Technology, China; Center for Translational Medicine, Wuhan Jinyintan Hospital, China; Joint Laboratory of Infectious Diseases and Health, Wuhan Institute of Virology and Wuhan Jinyintan Hospital, Wuhan Jinyintan Hospital, China; MOE Key Laboratory of Molecular Biophysics, Hubei Bioinformatics and Molecular Imaging Key Laboratory, Center for Artificial Intelligence Biology, College of Life Science and Technology, Huazhong University of Science and Technology, China; Department of Critical Care Medicine, Union Hospital, Tongji Medical College, Huazhong University of Science and Technology, China; Joint Laboratory of Infectious Diseases and Health, Wuhan Institute of Virology and Wuhan Jinyintan Hospital, Wuhan Jinyintan Hospital, China; Institute of Anesthesiology and Critical Care Medicine, Union Hospital, Tongji Medical College, Huazhong University of Science and Technology, China; State Key Laboratory of Virology, Wuhan Institute of Virology, Chinese Academy of Sciences, China; Joint Laboratory of Infectious Diseases and Health, Wuhan Institute of Virology and Wuhan Jinyintan Hospital, Wuhan Institute of Virology, Chinese Academy of Sciences, China; Center for Translational Medicine, Wuhan Jinyintan Hospital, China

Multi-organ injuries are a major complication of severe Coronavirus Disease 2019 (COVID-19). However, its pathogenesis is barely understood. Herein, we profile host responses to fatal SARS-CoV-2 infection by performing quantitative proteomics of 19 post-mortem samples of 8 different organs or tissues, including lungs, liver, intestine, kidney, spleen, brain, heart and muscle, from 3 deceased COVID-19 victims during the first wave of the pandemic (Supplementary Fig. S1A and B, Tables S1–S3). We analyzed the pulmonary autopsy specimens, and found that the main pathological change of COVID-19 in the lungs was diffuse alveolar damage (Supplementary Fig. S1C and D). The immunofluorescent staining of SARS-CoV-2 nucleocapsid protein (NP) confirmed the presence of SARS-CoV-2 antigens in COVID-19 lung tissues (Supplementary Fig. S1E). For the proteomic profiling, we used tandem mass tag (TMT) 11-plex labeling and liquid chromatography with tandem mass spectrometry (LC-MS/MS), while a pooling mixture of the 19 COVID-19 samples was used as an internal control to eliminate the batch effect (Tables S2 and S3).

From the LC-MS/MS analysis, we obtained 49 815 non-redundant peptides (Fig. 1A), which were unambiguously mapped to 5346 human proteins

quantified in at least one sample (Fig. 1B, Table S4). We evaluated the quality of the proteomic data by checking the original MS/MS data, and found that the average spectral count of all peptides was 2.66, with 29 618 peptides (59.5%) matched by ≥2 spectral counts. Thus, the results indicate that proteomic profiling is highly reliable at the peptide level. To enable protein expressions of all samples following a similar distribution, *z*-score transformation plus min-max- and median-centering normalizations were used to individually normalize the proteomic data for each sample, and the normalized protein expression (*NPE*) value was determined for each protein (Supplementary Fig. S2A, Table S5). Using *NPE* values of proteins, a principal component analysis (PCA) revealed that different COVID-19 post-mortem tissue types could be roughly separated (Supplementary Fig. S2B).

Next, we identified potential tissue-specific proteins (TSPs), and observed that different tissue types had distinct molecular signatures (Supplementary Figs S2C and D, S3 and S4, Table S6). For example, uromodulin (UMOD), a known kidney-specific protein, exhibited much higher expression in two kidney samples than other tissues (Supplementary Fig. S3). Also, S100A8 and its partner S100A9, two known acute-phase proteins, were only highly expressed in lung samples (Supplementary Fig. S3). In addition, Neurogranin (NRGN), a critical regulator in neurodevelopment, exhibited higher expression only in two brain samples (Supplementary Fig. S3). Thus, our proteomic profiling revealed a landscape of differential protein expression in COVID-19 post-mortem tissue types.

To identify differentially expressed proteins (DEPs) in COVID-19 post-mortem tissues, we downloaded the proteomic datasets of six normal human tissues from the Human Proteome Map, and the same *z*-score methods were used for data normalization (Supplementary Fig. S5A–F). Then, we used a tool called Model-Based Analysis of Proteomic data (MAP), and identified 2604, 611, 212, 173, 51 and 42 potential DEPs in the lungs, kidney, liver, intestine, brain and heart tissue samples, respectively (Fig. 1C–F, Supplementary Fig. S5G–I, Table S7, adjusted *P*-value < 0.05). We revealed numerous alterations of host proteins in different organs that might contribute to the pathogenesis of COVID-19 (Supplementary Fig. S6). For example, the protein levels of S100A8/A9, which have been found to be up-regulated in severe COVID-19 cases [1], were significantly elevated in lung tissues.

**Figure 1.**
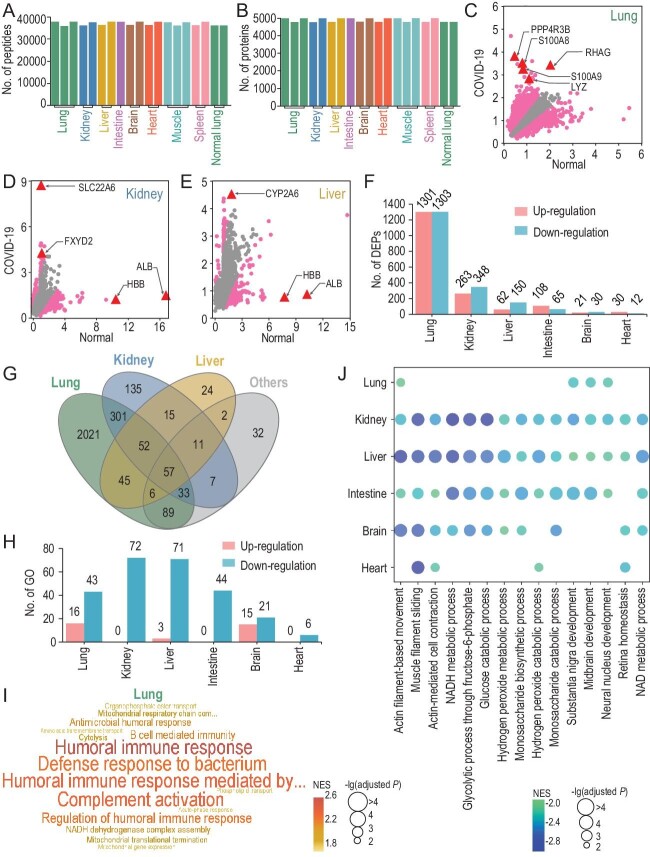
The multi-omic study of COVID-19 post-mortem tissue samples. (A and B) The distribution of numbers of quantified (A) peptides and (B) proteins in 17 post-mortem and 2 normal lung tissues. (C–E) MAP-based identification of potential DEPs in the (C) lung, (D) kidney and (E) liver of post-mortem tissues. Arrows indicate the DEPs in each tissue are also shown as TSPs. (F) The distribution of numbers of up- and down-regulated DEPs in the six types of post-mortem tissues. (G) The overlap of DEPs in different post-mortem tissues. (H) The distribution of numbers of significantly up- or down-regulated Gene Ontology (GO) biological processes detected by GSEA (adjusted *P*-value < 0.01) in the six types of post-mortem tissues. (I) Visualization of up-regulated processes in post-mortem lungs, using a word cloud illustrator WocEA. (J) Down-regulated processes in ≥4 post-mortem tissue types.

In particular, a number of TSPs such as S100A8/9, UMOD and NRGN were also significantly up-regulated in COVID-19 post-mortem tissues (Tables S6 and S7).

Moreover, many important proteins were down-regulated in different post-mortem tissues (Supplementary Fig. S6). For example, albumin (ALB) and hemoglobin (HBB), two fundamental proteins in plasma, were significantly

down-regulated in all six tissue types, especially in the liver and kidney (Fig. 1D and E, Supplementary Fig. S6B). Indeed, ALB and HBB have been proposed to be decreased in COVID-19 patients with more severe conditions [2]. Thus, our results not only identified DEPs markedly changed in different tissues, but also indicated the potential source organs responsible for their alternations in sera.

The count of potential DEPs across the six post-mortem tissue types demonstrated that lung tissues harbored the greatest number of DEPs (70.5%, 2604/3693) (Fig. 1G). In particular, overlaps were less observed through a comparison of different tissues, and 583 DEPs in the lungs were mutually shared by other tissues and 57 DEPs were shared by ≥4 tissues (Fig. 1G, Supplementary Fig. S6B), indicating that the lungs presented with the most significant protein alterations in response to COVID-19.

Using a tool called Gene Set Enrichment Analysis (GSEA), we identified 16, 3 and 15 biological processes markedly up-regulated in the lungs, liver and brain of COVID-19 tissues, respectively (Fig. 1H and I, Supplementary Fig. S7, Table S8). In post-mortem lung tissues, up-regulated processes were mainly focused on immune- and inflammation-related processes, which were not significantly elevated in other tissue types (Fig. 1I, Supplementary Fig. S7A and B, Table S8). Also, the cell morphology maintenance-related pathways, such as the endothelial barrier establishment, were only found to be down-regulated in lung tissues (Supplementary Fig. S7C), supporting the notion that the lungs are the major virus–host battlefield of COVID-19. In particular, we observed that many fundamental processes involved in organ movement, respiration and metabolism were dramatically down-regulated in the six post-mortem tissue types, with the number ranging from 6 (heart) to 72 (kidney) (Fig. 1I). In total, 15 basic processes, such as actin-filament-based movement (GO:0030048), Nicotinamide Adenine Dinucleotide Hydrate (NADH) metabolic process (GO:0006734) and glucose catabolic process (GO:0006007), were significantly down-regulated in ≥4 tissue types (Fig. 1J and Table S8). Based on these results, we propose that the brain and heart were less affected by COVID-19 in terms of the numbers of DEPs and altered processes (Supplementary Fig. S5H and I). Together, these results indicate that the responses of distinct COVID-19 tissues are different in critically ill conditions.

Moreover, we compared our data to the DEPs of different tissues from previously published proteomic studies (Supplementary Fig. S8, Table S9) [3–5]. In our data, Cathepsin L1 (CTSL) exhibited a 1.555-fold increase exclusively in COVID-19 lungs, which is consistent with Nie's data [3], but this is not shown in the human colon epithelial carcinoma cell line Caco-2 after SARS-CoV-2 infection [5]. Interestingly, a CTSL inhibitor could efficiently block the entry of SARS-CoV-2 into cells [6], indicating a potential contribution of CTSL to the pathogenesis of COVID-19. Moreover, we found that S100A8/A9 were significantly elevated in the lungs (Tables S5 and S9). However, neither of these two proteins was identified as a lung-specific DEP in Nie's work [3], whereas Leng *et al.* found that S100A9 was down-regulated in the lungs of COVID-19 patients (Table S9) [4]. Recently, Guo *et al.* validated the significant up-regulation of S100A8/A9 in animal models and patients [7], which supports our finding and highlights the contribution of S100A8/A9 to the pathogenesis of COVID-19. Together, ours and others’ findings support the proposition that the lungs are the major virus–host battlefield of COVID-19.

The emergence of new SARS-CoV-2 variants has caused global concern. Although the rapid spread of new SARS-CoV-2 strains indicates increased transmissibility, it is not clear whether infections are associated with alternation in pathogenicity compared to the previous SARS-CoV-2 strain. Thus, we obtained 48 DEPs from a recent multi-omic study of human lung epithelial cells infected with 3 new SARS-CoV-2 strains, including a lineage B isolate VIC, a lineage B.1.13 isolate IC19 and a lineage B.1.1.7 isolate Kent [8]. In our dataset, 18 of these DEPs were quantified in at least one tissue (Table S10). Among these DEPs, a number of fundamental proteins, such as ALB, α-2-macroglobulin (A2M) and α-2-HS-glycoprotein (AHSG), were all markedly down-regulated in our dataset and upon new strain infection, indicating that infections of both previous and new SARS-CoV-2 variants can cause strong deteriorative effects with regard to disrupting normal human functions. In contrast, a number of interferon-stimulated genes (ISGs), such as IFIT1, IFIT2, IFIT3 and ISG20, were significantly up-regulated upon new strain infection, but underwent mild or no change in our data (Table S10). Consistently, an enrichment analysis of the 48 DEPs found the type I interferon (IFN-I) signaling pathway significantly altered by the new strains (Table S11), indicating that immune responses can be induced by the new SARS-CoV-2 strains. On the other hand, a recent study uncovered that IFN-I response was highly activated in mild to moderate patients but diminished in severe ones [9]. In this current study, post-mortem tissues were taken from patients who were deceased from critical illness, and as expected, no, or few, IFN-I response-associated DEPs were identified. Therefore, although certain new SARS-CoV-2 strains have higher transmission and infection rates, it is hard to tell whether their physiological harm and ability to induce immune or inflammatory responses are alleviated or exacerbated; this should be answered by future post-mortem studies of the COVID-19 cases caused by the new SARS-CoV-2 variants.

Furthermore, for a better understanding of host responses in COVID-19 lungs, we modeled a network containing 110 known virus–host protein–protein interactions (PPIs) [10] between 23 SARS-CoV-2-encoded proteins and 110 interacting DEPs in the lungs (Supplementary Fig. S9, Table S12). The results demonstrated that at least six aspects, including immune response, metabolic process, transcription/translation, cell signaling/development, transport and cytoskeleton organization, were dysregulated by SARS-CoV-2 (Supplementary Figs S9 and S10, Table S13).

In summary, this post-mortem proteomic study reveals that COVID-19 is associated with extensive virus–host interactions and causes significant host responses in multiple organs, which contribute to multi-organ injuries. This work provides an invaluable proteome map and resource for understanding COVID-19-associated host responses, sheds light on the pathogenesis of COVID-19 and provides hints of potential therapy.

## Supplementary Material

nwab143_Supplemental_File
