# Supplementary material for: Post-mortem tissue proteomics reveals the pathogenesis of multi-organ injuries of COVID-19
Source: Natl Sci Rev. 2021 Aug 10;8(11):nwab143. doi: 10.1093/nsr/nwab143 (PMC8644997; doi:10.1093/nsr/nwab143)
Supplement: nwab143_Supplemental_File [file nwab143_supplemental_file.pdf]

Supplementary Materials for

Postmortem Tissue Proteomics Reveals the Pathogenesis of Multiorgan Injuries of COVID-19

Yang Qiu, Di Wu, Wanshan Ning, Jiqian Xu, Ting Shu, Muhan Huang, Rong Chen, Jiancheng Zhang, Yang Han, Qingyu Yang, Ruiting Li, Yuanyuan Bie, Xiaobo Yang, Yaxin Wang, Xiaojing Zou, Shangwen Pan, Chaolin Huang, Yu Xue, You Shang, Xi Zhou

Correspondence to: zhouxi@wh.iov.cn (X.Z.), you_shanghust@163.com (Y.S.), xueyu@hust.edu.cn (Y.X.), chaolin2020@163.com (C.H.)

This PDF file includes:

Supplementary results
Materials and Methods
References
Figs. S1 to S10

Other Supplementary Materials for this manuscript include the following:

Supplementary Table S1 to S13

Supplementary results

The experimental procedure of this study

Postmortem tissue samples were collected during the autopsy of 3 patients who were deceased from respiratory failure caused by SARS-CoV-2 infection at Wuhan Jinyintan Hospital. We collected the samples of lung and muscle from Patient 1, the samples of lung, heart, liver, spleen, kidney, intestine, brain and muscle from Patient 2, and the samples of lung, heart, liver, spleen, kidney, brain and muscle from Patient 3 (Supplementary Fig. S1A, Table S1-S3). Besides, lung paracancerous tissue samples from two lung cancer patients were collected for comparison. For each tissue sample, total proteins were extracted and processed by trypsin, and the resulting peptides were subjected to tandem mass tag (TMT) 11-plex labeling (Supplementary Fig. S1B). The peptide samples were individually labeled and analyzed in 2 batches by using liquid chromatography with tandem mass spectrometry (LC-MS/MS) (Supplementary Fig. S1B). To eliminate the batch effect, the pooling mixture of the 19 samples was used as an internal control for each batch, and allocation of the 19 samples was completely random (Table S2 and S3).

Prior to the proteomic profiling, we analyzed the pathology of pulmonary autopsy specimens from patients 2 and 3. The main pathological change of the post-mortem lung tissues from two patients was diffuse alveolar damage (Supplementary Fig. S1C), which is similar with that caused by SARS-CoV [1]. The histology was represented mainly by a widespread destruction of pulmonary architecture, with extensive fibromyxoid exudate, alveolar haemorrhage, formation of hyaline membranes, and interstitial thickening. In addition, the ultrastructure of these lung tissue samples under transmission electron microscopy revealed several virion-like particles in alveolar epithelial cells (Supplementary Fig. S1D). These virion-like particles were approximately 80-120 nm in diameter, with spiky-like projections on the surface and typical electron lucent center, which display typical coronavirus morphology of SARS-CoV-2 virion [2]. Furthermore, the immunofluorescent staining assays were performed to detect the presence of SARS-CoV-2 nucleocapsid protein (NP) in lung tissue samples (Supplementary Fig. S1E).

A protein atlas of eight COVID-19 postmortem tissue types

From the LC-MS/MS analysis, we obtained 49,815 non-redundant peptides, with a number ranged from 36,046 to 37,855 peptides in 3 lung, 2 kidney, 2 liver, 1 intestine, 2 brain, 2 heart, 3 muscle and 2 spleen samples of COVID-19 postmortem tissues, as well as 2 normal lung samples

(Fig. 1A). These peptides were mapped to their corresponding protein sequences, and we used the reporter ion MS2 module of the MaxQuant software package for protein quantification [3]. From the results, we observed that 5346 human proteins were quantified in at least one sample (Table S4), with protein numbers ranged from 4776 to 5000 (Fig. 1B). The protein coverage of using TMT-labeling strategy on multiple samples is expected to be lower compared to that on single sample, because the complexity is increased due to the mixture of multiple samples. Both human and SARS-CoV-2 protein sequences were included for database search, while no viral proteins were detected in any tissue samples, probably due to the background of large amount of host proteins.

After data normalization, we obtained the normalized protein expression (*NPE*) values of proteins (Table S5). Then, we used an entropy-based method [4, 5] to identify 226 potential tissue-specific proteins (TSPs), including 158 TSPs in brain and 68 TSPs in other tissues, respectively (Supplementary Figs. S2C, S2D, S3 and S4, Table S6). This result is consistent with the existing knowledge, since brain is one of the most specialized organs in the human body. Thus, it’s not surprised that brain has most potential TSPs. Also, a hierarchical clustering was conducted for all proteins in the eight tissue types, and the result was visualized by a software package named Heatmap Illustrator (HemI) [6]. Obviously, different tissue types had distinct molecular signatures, and potential TSPs can be directly recognized from the heatmap (Supplementary Fig. 2D). Based on the annotations of GeneCards (https://www.genecards.org/) [7], a comprehensive database for human genes, several TSPs were picked out and shown for each tissue sample (Supplementary Figs. S3 and S4).

Proteomic alterations reveal that human tissues are differentially affected in response to COVID-19

To probe the protein changes upon SARS-CoV-2 infection, we downloaded the proteomic datasets of six normal human tissues from the Human Proteome Map (HPM) [8], with a number of quantified proteins ranged from 12,007 to 16,868 (Supplementary Fig. S5A). Compared to HPM, > 96.0% of proteins quantified in this study were covered by HPM (Supplementary Fig. S5B). To enable an unbiased comparison between COVID-19 and normal samples, the same *z*-score plus min-max and median centering methods were used to individually normalize each dataset (Supplementary Fig. S5C,). The distribution of original COVID-19 protein expressions

and HPM data before normalization and the *NPE* values after normalization were shown in Supplementary Figure S3D and S3E. We showed that the protein expressions of all datasets were normalized and normally distributed. Moreover, the PCA analysis demonstrated that COVID-19 and normal tissues could be unambiguously separated, irrespective of the data source (Supplementary Fig. S3F).

To identify differentially expressed proteins (DEPs), we used a tool named Model-based Analysis of Proteomic data (MAP) to analyze each pair of COVID-19 and normal tissues [9]. Muscle and spleen samples were not analyzed due to the lack of the corresponding normal tissues data in HPM. In contrast with conventional statistical methods, MAP did not estimate technical and systematic errors from technical replicates. Based on a hypothesis that technical and systematic errors might be approximately identical for quantified proteins within a small window, the standard normal distribution was adopted to model the proteomic data and directly calculate a *p*-value for each protein (Table S7).

A COVID-19-associated protein-protein interaction network

We mapped the protein-protein interactions between SARS-CoV-2-encoded proteins and DEPs by using a published interactome data of SARS-CoV-2 proteins [10]. We obtained 110 known virus-host protein-protein interactions (PPIs) between 23 viral proteins and 110 interacting DEPs differentially regulated in postmortem lung tissues (Table S12). Other lung DEPs were also included for modeling an integrative virus-host molecular network. These interacting DEPs were classified into 6 groups according to their functions, including immune response, metabolic process, transcription/translation, cell signaling/development, transport, and cytoskeleton organization, which are participate in almost all the major biological functions in host (Supplementary Fig. S9). Moreover, Gene Ontology (GO) analysis showed that these DEPs were generally involved in several immune response-related processes, including Rab protein signal transduction, blood coagulation and neutrophil degranulation (Supplementary Fig. S9 and Table S13), which are consistent with the previous findings that cytokine storm, alveolar macrophage activation, intravascular coagulation and microthrombosis are frequently presented in severe COVID-19 cases [11, 12]. Together, these results suggest that SARS-CoV-2-encoded proteins might affect the functions of the interacting host proteins in infected lungs.

Supplementary discussion

COVID-19 is caused by SARS-CoV-2, which is the third coronavirus to cause severe respiratory disease in humans besides SARS-CoV and Middle East respiratory syndrome coronavirus (MERS-CoV). Since its emergence from late 2019 [13], the outbreak of SARS-CoV-2 has resulted in tremendous impacts on global health, social and economics, making COVID-19 a global pandemic and the worst public health crisis once a century.

About 20% COVID-19 patients have been reported to develop severe or critical conditions [14], and the mortality rate of critically ill cases can reach over 60% [15]. The main targets of SARS-CoV-2 are human low respiratory tract and lung, while many other organs, including liver, heart, intestine, kidney, central nervous system and muscle have been also found to be injured [16-18]. Among the broad symptoms of COVID-19, fever, pneumonia, respiratory failure, acute respiratory distress syndrome (ARDS),

and sepsis are frequently observed complications, which are usually associated with pathophysiological changes such as alveolar macrophage activation, lymphopenia, cytokine release syndrome, thrombosis and intravascular coagulation in severe COVID-19 patients [11, 13, 19-23]. However, despite of extensive efforts made by global scientific community to study this emerging coronavirus disease, the molecular mechanisms underlying its pathogenesis, particularly the pathogenesis of COVID-19-associated multiorgan injuries, are still barely understood, which represents a major obstacle to fully understand and find out effective ways to combat against this deadly coronavirus disease. In this study, we provide the postmortem tissue proteomic datasets that provides the most direct and reliable evidence of the pathophysiological changes of human bodies in response to SARS-CoV-2 infections, and uncovers that SARS-CoV-2 infection affected different set of host processes in different organs or tissues, which probably contribute to the pathogenesis of COVID-19-associated multiorgan injuries.

One of the key findings obtained here is that proteins and pathways are differently altered in distinct human tissues or organs in response to COVID-19. In a recent study, Nie et al. identified that immune- and inflammation-related pathways were significantly up-regulated in multiple organs, such as lung, spleen, heart, kidney, and thyroid [24]. However, our analysis found that these processes, such as humoral immune response, complement activation, B-cell mediated immunity, acute phase response and cytolysis, were upregulated only in lungs from all the tissues examined, showing that excessive immune response and inflammation were extensively occurred

in lungs. Consistently, our histopathological examinations also showed that interstitial mononuclear/macrophage cell infiltration and inflammation were presented in lung tissues. On the other hand, cell morphology maintenance-related pathways were downregulated in lungs. These results indicated that the microscopic structure of alveolar cells and lungs were severely damaged, consistent with the postmortem pathological and histopathological observations in the current study and by others that extensive fibromyxoid exudation, alveolar haemorrhage and thrombosis were found in lungs [25, 26]. Therefore, we conclude that the excessive inflammation in lungs of severe COVID-19 cases increases vascular permeability and activates coagulation cascades, resulting in vascular thrombosis and probably a systemic hypoxia, and also causes a widespread destruction of pulmonary architecture and functions. Our findings are in accordance with previous clinical and autopsy observations that severe or critical ill COVID-19 patients are frequently associated with massive intravascular thrombus, hypoxemia, and ARDS [13, 15, 17, 19-22, 25-27], which are pathophysiologically associated with cytokine storm, alveolar macrophage activation, intravascular coagulation and microthrombosis [11, 12, 23].

On the other hand, unlike the host protein responses in lungs, our study revealed that the DEPs in tissues of liver, kidney, intestine, brain, and heart are mainly present in pathways involved in organ movement, respiration, and metabolism. For example, some shared altered pathways, including muscle filament sliding and contraction, cellular respiration, NADH metabolic process, hydrogen peroxide metabolic process, and glucose catabolic process, were found to be significantly downregulated in kidney, liver, intestine, and brain, and these findings were highly consistent with the Nie's study [24]. These results indicated that these tissues were affected by hypoxia and their functions and morphology were dramatically impaired, which are consistent with the previous clinical data that multiorgan failure are frequently observed complications in severe COVID-19 cases [13, 15, 20, 21]. Surprisingly, based on our proteomic data, very few immune- or inflammation-related pathways were found to be significantly altered in other organs/tissues, indicating that the leading cause of multiorgan injuries in non-lung organs/tissues is hypoxia but not excessive inflammation. Thus, we propose that lung is the center of the virus-host battlefields of COVID-19, and the excessive inflammatory responses to SARS-CoV-2 infection in lungs result in the thrombosis and destruction of pulmonary architecture and functions, leading to hypoxia of multiple organs in the whole body and subsequent disease aggravation.

Omics studies under the pathophysiological conditions caused by viral infections are powerful weapons to explore the pathogenesis of viral infectious diseases, establish animal models as well as develop potential clinical treatments. After the outbreak of COVID-19, both direct RNA sequencing (DRS)-based transcriptomic and LC-MS/MS-based proteomic, metabolomic or lipidomic profilings were conducted for analyzing SARS-CoV-2 and/or host samples [15, 24, 28-35]. Particularly, Gordon et al. generated a SARS-CoV-2-encoded protein interactome using affinity-purification mass spectrometry (AP-MS) [10]. In this study, using this interactome data, we generated 110 known virus-host PPIs between 22 viral proteins of SARS-CoV-2 and 110 interacting DEPs in lung tissues, suggesting that these viral proteins directly affect the expressions and/or functions of these interacting host proteins. Therefore, it would be intriguing to integrate the omics data to generate a more comprehensive picture of the pathogenicity of SARS-CoV-2 and the pathogenesis of COVID-19.

Taken together, our findings demonstrate the significant pathophysiological alternations of host proteins/pathways associated with multiorgan injuries of COVID-19, which provides invaluable knowledge about COVID-19-associated host responses and sheds light on the pathogenesis of COVID-19.

Supplementary Methods

Ethics and Human Subjects

All work performed in this study was approved by the Wuhan Jinyintan Hospital Ethics Committee (No. KY-2020-15.01). Diagnosis of SARS-CoV-2 infection was based on the New Coronavirus Pneumonia Prevention and Control Program (6th edition) published by the National Health Commission of China.

Patient and Samples

We analyzed postmortem tissue samples from 3 patients who died from respiratory failure caused by SARS-CoV-2 infection at Wuhan Jinyintan Hospital. Briefly, Patient 1 is a 53-year-old female. Patient 2 is a 62-year-old male. Patient 3 is a 66-year-old female (Table S1). All the patients had fever, cough and shortness of breath and progressed into ARDS due to severe pulmonary lesions with significantly decreased lymphocytes. Finally, all these patients died of respiratory failure.

We collected the samples of lung and muscle from patient 1, the samples of lung, heart, liver, spleen, kidney, intestine, brain and muscle from patient 2, and the samples of lung, heart, liver, spleen, kidney, brain and muscle from patient 3. Besides, lung paracancerous tissue samples from 2 lung cancer patients (a 65-year-old male and a 57-year-old female) were collected for comparison (Table S1). For all the three deceased COVID-19 patients, the interval time between patient decease and autopsy was less than 1 h, and postmortem specimens were immediately frozen in liquid nitrogen after dissection. All the samples were treated according to the biocontainment procedures of the processing of SARS-CoV-2-positive sample. For the cancer patients, the paracancerous tissue samples were resected from the patients and also immediately stored in liquid nitrogen before further treatment.

Haematoxylin and eosin staining and immunofluorescence analysis

Tissues from the case were fixed with 4% paraformaldehyde for 24 h. Tissues were then embedded in optimal cutting temperature (OCT) compound and cut into 3.5-μm sections using Rotary Microtome (Thermo Scientific™ HM 355S). Mounted microscope slides were fixed with paraformaldehyde and stained with haematoxylin and eosin for histopathological examination.

Slides were dewaxed with dimethylbenzene and gradient alcohol, antigen repaired with ethylene diamine tetraacetic acid (pH=8.0), then blocked by incubating with 5% bovine serum albumin (BIOSHARP, Hefei, China) at 37 ℃ for 30 min, followed by overnight incubation at 4 ℃ with the rabbit anti- SARS-CoV-2 nucleocapsid protein (NP) antibody (1:200) [2] in phosphate buffered solution. After washing, slides were then incubated for 1 h at room temperature with fluorescein isothiocyanate-conjugated goat-anti-rabbit IgG (Proteintech) in PBS, then stained with 2-(4-Amidinophenyl)-6-indolecarbamidine dihydrochloride (DAPI, Beyotime, Nanjing, China) and observed under a fluorescence microscope (Nikon A1 MP STORM).

<u>Transmission electron microscopy</u>

Tissues from the case were fixed with 2.5% (weight/volume) glutaraldehyde, post-fixed with 1% osmium tetroxide, and then dehydrated with gradient alcohol (from 30%-100%), embedded with epoxy resin. Ultrathin sections (80 nm) of embedded cells were prepared, deposited onto Formvar-coated copper grids (200 mesh), double-stained with uranium acetate and lead citrate, then observed under 200 kV Tecnai G2 electron microscope (ThermoFisher Scientific FEI).

<u>Sample preparation</u>

The tissue samples were first homogenized in lysis buffer consisted of 2.5% SDS/100 mM Tris-HCl (pH 8.0) [36]. The wet weight, protein concentration and total protein weight for each tissue sample are shown in Table S2. After 15 min of incubation in the boiling water bath, the samples were subjected to treatment with ultra-sonication. After centrifugation (12000 × *g*, 15 min), proteins in the supernatant were precipitated by adding 4 times of cold acetone. The protein sample was dissolved in 8 M Urea/100 mM Tris-HCl (pH 8.0). After centrifugation, the supernatant was used for reduction reaction (10 mM DTT, 37 ℃ for 1 h), and followed by alkylation reaction (40 mM iodoacetamide, room temperature/dark place for 30 min). Protein concentration was measured by Bradford method. Urea was diluted below 2 M using 100 mM Tris-HCl (pH 8.0). Trypsin was added at a ratio of 1:50 (enzyme: protein, *w*/*w*) for overnight digestion at 37 ℃. The next day, trifluoroacetic acid (TFA) was used to bring the pH down to 6.0 to end the digestion. After centrifugation, the supernatant was subjected to peptide purification using Sep-Pak C18 desalting column. The peptide eluate was dried in vacuum and stored at -20 ℃ for later use.

Tandem mass tag (TMT) labeling was performed according to manufacturer's instructions. Briefly, peptides were reconstituted in TMT reagent buffer, and the samples were separately labeled with different TMT labeling reagents. The labeled samples were then mixed and subjected to Sep-Pak C18 desalting. The complex mixture was fractionated using high pH reversed-phase liquid chromatography (RPLC) and combined into 20 fractions. Each fraction was dried in vacuum and stored at -80 ℃ until MS analysis.

LC-MS/MS analysis

LC-MS/MS data acquisition was carried out on a Q Exactive HF-X mass spectrometer coupled with an Easy-nLC 1200 system (both Thermo Scientific). Peptides were first loaded onto a C18 trap column (75 μm × 2 cm, 3 μm particle size, 100 Å pore size, Thermo) and then separated in a C18 analytical column (75 μm × 250 mm, 3 μm particle size, 100 Å pore size, Thermo). Mobile phase A (0.1% formic acid) and mobile phase B (80% ACN, 0.1% formic acid) were used to establish the separation gradient. The total collection time of each TMT batch mass spectrum is 20 h. Each batch is divided into 20 components and subjected to 60 min LC gradient per fraction. A constant flow rate was set at 300 nL/min. For data-dependent acquisition (DDA) mode analysis, each scan cycle consisted of one full-scan mass spectrum (R = 120 K, AGC = 3e6, max IT = 50 ms, scan range = 350–1800 m/z) followed by 20 MS/MS events (R = 45 K, AGC = 1e5, max IT = 86 ms). High energy collision dissociation (HCD) collision energy was set to 32. Isolation window for precursor selection was set to 1.2 Da. Former target ion exclusion was set for 45 s.

Database research

MS raw data were analyzed with MaxQuant (V1.6.6) using the Andromeda database search algorithm. The human proteome database contained 20,366 Swiss-Prot/reviewed human protein sequences downloaded from UniProt (https://www.uniprot.org/proteomes/UP000005640, on March 17, 2020), whereas the SARS-CoV-2 proteome database contained 12 protein sequences derived from its CDS regions (https://www.ncbi.nlm.nih.gov/nuccore/NC_045512.2, on March 17, 2020) [37]. The two databases were merged and reverse decoy sequences were generated. Then, spectra files were searched against the merged database using the following parameters: Type, TMT; Variable modifications, Oxidation (M), Deamidation (NQ), Acetyl (Protein N-term); Fixed modifications, Carbamidomethyl (C); Digestion, Trypsin/P. The MS1 match tolerance was set as

20 ppm for the first search and 4.5 ppm for the main search. The MS2 tolerance was set as 20 ppm. Search results were filtered with 1% false discovery rate (FDR) at both protein and peptide levels. Proteins denoted as decoy hits, contaminants, or only identified by sites were removed, and the remaining proteins were used for further analysis.

Proteomic data imputation and normalization

To ensure the data quality, only 4993 proteins quantify in ≥ 10 samples were reserved. The missing values were imputed with values representing a normal distribution around the detection limit of the mass spectrometer. For each sample, the mean and standard deviation (S.D.) of the distribution of the raw protein intensities were determined. Then a new distribution with a downshift of 1.5 S.D. and a width of 0.3 S.D. was created. The total data set was imputed using these values, enabling statistical analysis.

After imputation, the intensity-based expression value of a protein was first normalized based on its expression level in the control sample of the same batch to eliminate the batch effect. Then, the proteomic data of each sample was normalized into a similar distribution using the *z*-score transformation, one of the mostly used normalization methods. For each sample, the mean expression value μ and S.D. δ were first calculated. For a protein *i* with the expression level of x_i, its normalized *z*-score was calculated as below:

$$z_i = \frac{x_i - \mu}{\delta}$$

For each z_i, we re-scaled it into a value ranged from 0 to 1 by min-max normalization shown as below:

$$z_i^* = \frac{z_i - Min}{Max - Min}$$

Where *Max* and *Min* were maximum and minimum expression values in the sample. The median centering method was further used, and the *NPE* value for *i* was calculated as below:

$$NPE_i = \frac{z_i^*}{Median}$$

After *z*-score coupled with min-max and median centering normalizations, *NPE* values of proteins follow a logarithmic normal distribution ($\log_2$) centered at zero.

The proteomic data imputation and normalization was conducted using Perseus 1.6.14 [3]. To test whether different tissue-specific proteomes could be distinguished, PCA was performed

using Scikit-learn 0.22.1 (https://scikit-learn.org/stable/), a powerful toolkit for data mining and analysis.

Human normal proteomic data

From the HPM portal (http://humanproteomemap.org/) [8], pre-compiled proteomic datasets of lung, kidney, liver, colon, frontal cortex and heart in adults were downloaded, containing 12,335, 12,252, 16,800, 14,813, 16,868, and 12,007 quantified proteins, respectively. The HPM proteomic datasets were also imputed and normalized by the same methods described above.

Identification of tissue-specific proteins

As previously described [4, 5], an entropy-based method was adopted to identify potential TSPs in human COVID-19 tissues. For each protein, its relative *NPE* (*rNPE*) value in a tissue *j* was defined as below:

$$rNPE_j = \frac{NPE_j}{\sum_1^N NPE}$$

Where NPE_j was the normalized expression value in the tissue *j*, and Σ *NPE* was the sum of all *NPE* values in all tissue samples. *N* was the total number of COVID-19 tissues. Then, the Shannon entropy *H* of this protein across different tissues could be calculated as below:

$$H = -\sum_1^N rNPE_j \times log_2(rNPE_j)$$

Where the value of *H* ranged from 0 to $\log_2(N)$. A smaller *H* score represented a higher probability of a protein to be a real TSP. Based on the distribution of *H* scores, proteins with entropy < 2.5 were reserved as potential TSPs.

Model-based identification of differentially expressed protein (DEPs)

In this study, MAP was directly used to identify potential DEPs for each pair of COVID-19 and normal tissues [8]. For a two-sample comparison, MAP first ranks all proteins based on their $\log_2$-intensity changes. Then, MAP estimates local technical and systematic errors for each small interval by considering the changes of all proteins in the interval, and significantly altered proteins can be detected. Thus, MAP does not require technical replicates, which are commonly used for estimation of the same parameters. For a tissue type with multiple samples, the mean *NPE* value

was calculated for each protein. Then, the $\log_2$ ratios of COVID-19 *vs*. normal tissues for all proteins were determined and ordered based on their values. For using MAP, default parameters were adopted, with a sliding window size of 400 proteins, a step size of 100 proteins, and a fraction of 50 proteins [8]. In MAP, the Benjamini–Hochberg method was used for adjustment of multiple testing, and an adjusted p-value < 0.05 was selected to identify potential DEPs. Fold changes of postmortem *vs*. normal tissues were also present for identified DEPs.

GSEA enrichment analysis

The software package of GSEA v4.0.3 was downloaded (https://gsea-msigdb.org) [38], as well as the gene set collection of GO biological processes with gene symbols (c5.bp.v7.1.symbols.gmt). A stringent threshold of FDR q-val < 0.01 was adopted to detect GO biological processes significantly up- or down-regulated in COVID-19 tissues.

Re-construction of a virus-host protein interaction network

From the Human Protein Atlas (HPA), we obtained 331 known virus-host PPIs (https://www.proteinatlas.org/humanproteome/sars-cov-2) reported by a recent study [10]. Since lung is the potentially major virus-host battlefield of COVID-19, only 110 virus-host PPIs were reserved for 23 SARS-CoV-2 proteins/mature peptides and 110 interacting DEPs in postmortem lung tissues. The 198 DEPs of 16 up-regulated biological processes in COVID-19 lung tissues were also included. Based on the functional annotations in UniProt, we classified the 308 lung DEPs into 6 classes, including cell signaling/development, cytoskeleton organization, immune response, metabolic process, transcription/translation, and transport. Then, 1,771,193 human known PPIs of 18,839 proteins were integrated from 7 public databases, including BioGrid [39], IID [40], InBio Map™ [41], Mentha [42], HINT [43], iRefIndex [44] and PINA [45]. For the 308 lung DEPs, we extracted 2,478 PPIs for 298 unique proteins, and the virus-host protein interaction network was constructed and visualized with Cytoscape 3.6.1 software package [46].

GO enrichment analyses

The two-sided hypergeometric test was adopted for the enrichment analysis of the 110 SARS-CoV-2 interacting DEPs. Here, we defined:

N = number of human proteins annotated by at least one term

n = number of human proteins annotated by term t

M = number of the 110 DEPs by at least one term

m = number of the 110 DEPs annotated by term t

Then, the E-ratio was calculated, and the P value was computed with the hypergeometric distribution as below:

$$\text{E-ratio} = \frac{\frac{m}{M}}{\frac{n}{N}}$$

$$P \text{ value} = \sum_{m'=m}^{n} \frac{\binom{M}{m'}\binom{N-M}{n-m'}}{\binom{N}{n}}, \text{ (E-ratio > 1)}$$

In this study, GO annotation files (on 03 January 2020) were downloaded from the Gene Ontology Consortium Web site (http://www.geneontology.org/), and we obtained 19,714 human proteins annotated with at least one GO biological process term.

Comparison of DEPs identified from other proteomic studies

Prior to our study, there were 7 omics studies of host protein changes upon SARS-CoV-2 infection, including 5 in cell lines, 1 in lung tissues, and 1 in 144 autopsy samples from 7 organs. These works included: 1) Bojkova et al. used the stable isotope labeling by amino acids in cell culture (SILAC) labeling and quantified 6385 proteins in the human colon epithelial carcinoma cell line Caco-2 with or without SARS-CoV-2 infection [28]; 2) Bezstarosti et al. used the label-free technique and quantified 6503 proteins from the African green monkey kidney Vero E6 cells with or without SARS-CoV-2 infection [32]; 3) Grenga et al. used the label-free technique and quantified 3320 proteins from Vero cells with or without SARS-CoV-2 infection [34]; 4) Appelberg et al. used the TMT labeling and quantified 7757 proteins in the human hepatocyte-derived cellular carcinoma cell line Huh7 with or without SARS-CoV-2 infection [35]; 5) Leng et al. obtained 3 lung tissue samples from 2 COVID-19 patients, and quantified 3321 proteins using the label-free technique [31]; 6) Nie et al. used TMT 16-plex labeling and quantified 11,394 proteins from 144 autopsies of 7 organs including lung, spleen, liver, kidney, heart, testis and thyroid in 19 COVID-19 patients [24]; 7) Thorne et al. conducted transcriptomic, proteomic and phosphoproteomic quantifications of human airway epithelial Calu-3 cells infected by 3 new SARS-CoV-2 strains, including the B lineage isolate BetaCoV/Australia/VIC01/2020 (VIC), B.1.13 lineage isolate hCoV-19/England/IC19/2020 (IC19), and B.1.1.7 lineage isolate hCoV-

19/England/204690005/2020 B (Kent), for 10 or 24 h [47]. Calu-3 cells infected with empty vector were taken as the mock control. On average, the numbers of identified proteins ranged from 3600 to 4000.

From Leng's study, we obtained 641 pre-determined DEPs in lung with COVID-19 [31]. From Bojkova's paper, we obtained 2734 DEPs from Caco-2 cells after SARS-CoV-2 infection [32]. From Nie's paper, we obtained 1606, 1585, 642, 1969, and 919 DEPs from lung, renal cortex, renal medulla, liver, and heart of COVID-19 patients [24]. From Thorne's study, we obtained 48 non-redundant DEPs in new virus strains against mock or between different new strains, while DEPs in Mock_10h against Mock_24h were not considered [47].

Data and Software Availability

The mass spectrometry proteomics data have been deposited to the ProteomeXchange Consortium (http://proteomecentral.proteomexchange.org) via the iProX partner repository with the dataset identifier PXD019970.

Supplementary References

1. Ding, Y, Wang, H, Shen, H, *et al.* The clinical pathology of severe acute respiratory syndrome (SARS): a report from China. *J Pathol*. 2003; **200**(3): 282-9.
2. Zhou, P, Yang, XL, Wang, XG, *et al.* A pneumonia outbreak associated with a new coronavirus of probable bat origin. *Nature*. 2020; **579**(7798): 270-3.
3. Tyanova, S, Temu, T, Cox, J. The MaxQuant computational platform for mass spectrometry-based shotgun proteomics. *Nat Protoc*. 2016; **11**(12): 2301-19.
4. Schug, J, Schuller, WP, Kappen, C, *et al.* Promoter features related to tissue specificity as measured by Shannon entropy. *Genome Biol*. 2005; **6**(4): R33.
5. Xie, W, Schultz, MD, Lister, R, *et al.* Epigenomic analysis of multilineage differentiation of human embryonic stem cells. *Cell*. 2013; **153**(5): 1134-48.
6. Deng, W, Wang, Y, Liu, Z, *et al.* HemI: a toolkit for illustrating heatmaps. *PLoS One*. 2014; **9**(11): e111988.
7. Stelzer, G, Rosen, N, Plaschkes, I, *et al.* The GeneCards Suite: From Gene Data Mining to Disease Genome Sequence Analyses. *Curr Protoc Bioinformatics*. 2016; **54**: 1 30 1-1 3.
8. Kim, MS, Pinto, SM, Getnet, D, *et al.* A draft map of the human proteome. *Nature*. 2014; **509**(7502): 575-81.
9. Li, M, Tu, S, Li, Z, *et al.* MAP: model-based analysis of proteomic data to detect proteins with significant abundance changes. *Cell Discov*. 2019; **5**: 40.
10. Gordon, DE, Jang, GM, Bouhaddou, M, *et al.* A SARS-CoV-2 protein interaction map reveals targets for drug repurposing. *Nature*. 2020; **583**(7816): 459-68.
11. Moore, JB, June, CH. Cytokine release syndrome in severe COVID-19. *Science*. 2020; **368**(6490): 473-4.
12. Subbarao, K, Mahanty, S. Respiratory Virus Infections: Understanding COVID-19. *Immunity*. 2020; **52**(6): 905-9.
13. Huang, C, Wang, Y, Li, X, *et al.* Clinical features of patients infected with 2019 novel coronavirus in Wuhan, China. *Lancet*. 2020; **395**(10223): 497-506.
14. The Novel Coronavirus Pneumonia Emergency Response Epidemiology, T. The Epidemiological Characteristics of an Outbreak of 2019 Novel Coronavirus Diseases (COVID-19) — China, 2020. *China CDC Weekly*. 2020; **2**(8): 113-22.
15. Kim, D, Lee, JY, Yang, JS, *et al.* The Architecture of SARS-CoV-2 Transcriptome. *Cell*. 2020; **181**(4): 914-21 e10.
16. Zhang, C, Shi, L, Wang, FS. Liver injury in COVID-19: management and challenges. *Lancet Gastroenterol Hepatol*. 2020; **5**(5): 428-30.
17. Varga, Z, Flammer, AJ, Steiger, P, *et al.* Endothelial cell infection and endotheliitis in COVID-19. *Lancet*. 2020; **395**(10234): 1417-8.
18. De Felice, FG, Tovar-Moll, F, Moll, J, *et al.* Severe Acute Respiratory Syndrome Coronavirus 2 (SARS-CoV-2) and the Central Nervous System. *Trends Neurosci*. 2020; **43**(6): 355-7.
19. Wang, D, Hu, B, Hu, C, *et al.* Clinical Characteristics of 138 Hospitalized Patients With 2019 Novel Coronavirus-Infected Pneumonia in Wuhan, China. *JAMA*. 2020; **323**(11): 1061-9.
20. Guan, WJ, Ni, ZY, Hu, Y, *et al.* Clinical Characteristics of Coronavirus Disease 2019 in China. *N Engl J Med*. 2020; **382**(18): 1708-20.
21. Chen, N, Zhou, M, Dong, X, *et al.* Epidemiological and clinical characteristics of 99 cases of 2019 novel coronavirus pneumonia in Wuhan, China: a descriptive study. *Lancet*. 2020; **395**(10223): 507-13.

22. Yang, X, Yang, Q, Wang, Y, *et al.* Thrombocytopenia and its association with mortality in patients with COVID-19. *J Thromb Haemost*. 2020; **18**(6): 1469-72.
23. Jose, RJ, Manuel, A. COVID-19 cytokine storm: the interplay between inflammation and coagulation. *Lancet Respir Med*. 2020; **8**(6): e46-e7.
24. Nie, X, Qian, L, Sun, R, *et al.* Multi-organ proteomic landscape of COVID-19 autopsies. *Cell*. 2021; **184**(3): 775-91 e14.
25. Ackermann, M, Verleden, SE, Kuehnel, M, *et al.* Pulmonary Vascular Endothelialitis, Thrombosis, and Angiogenesis in Covid-19. *N Engl J Med*. 2020; **383**(2): 120-8.
26. Xu, Z, Shi, L, Wang, Y, *et al.* Pathological findings of COVID-19 associated with acute respiratory distress syndrome. *Lancet Respir Med*. 2020; **8**(4): 420-2.
27. Ranucci, M, Ballotta, A, Di Dedda, U, *et al.* The procoagulant pattern of patients with COVID-19 acute respiratory distress syndrome. *J Thromb Haemost*. 2020; **18**(7): 1747-51.
28. Bojkova, D, Klann, K, Koch, B, *et al.* Proteomics of SARS-CoV-2-infected host cells reveals therapy targets. *Nature*. 2020; **583**(7816): 469-72.
29. Shen, B, Yi, X, Sun, Y, *et al.* Proteomic and Metabolomic Characterization of COVID-19 Patient Sera. *Cell*.
30. Wu, D, Shu, T, Yang, XB, *et al.* Plasma metabolomic and lipidomic alterations associated with COVID-19. *National Science Review*. 2020; **7**(7): 1157-68.
31. Leng, L, Cao, R, Ma, J, *et al.* Pathological features of COVID-19-associated lung injury: a preliminary proteomics report based on clinical samples. *Signal Transduct Target Ther*. 2020; **5**(1): 240.
32. Bezstarosti, K, Lamers, MM, van Kampen, JJA, *et al.* Targeted proteomics as a tool to detect SARS-CoV-2 proteins in clinical specimens. *bioRxiv*. 2020: 2020.04.23.057810.
33. Shu, T, Ning, W, Wu, D, *et al.* Plasma Proteomics Identify Biomarkers and Pathogenesis of COVID-19. *Immunity*. 2020; **53**(5): 1108-22 e5.
34. Grenga, L, Gallais, F, Pible, O, *et al.* Shotgun proteomics analysis of SARS-CoV-2-infected cells and how it can optimize whole viral particle antigen production for vaccines. *Emerg Microbes Infect*. 2020; **9**(1): 1712-21.
35. Appelberg, S, Gupta, S, Svensson Akusjärvi, S, *et al.* Dysregulation in Akt/mTOR/HIF-1 signaling identified by proteo-transcriptomics of SARS-CoV-2 infected cells. *Emerg Microbes Infect*. 2020; **9**(1): 1748-60.
36. Miao, M, Yu, F, Wang, D, *et al.* Proteomics Profiling of Host Cell Response via Protein Expression and Phosphorylation upon Dengue Virus Infection. *Virol Sin*. 2019; **34**(5): 549-62.
37. Wu, F, Zhao, S, Yu, B, *et al.* A new coronavirus associated with human respiratory disease in China. *Nature*. 2020; **579**(7798): 265-9.
38. Subramanian, A, Tamayo, P, Mootha, VK, *et al.* Gene set enrichment analysis: a knowledge-based approach for interpreting genome-wide expression profiles. *Proc Natl Acad Sci U S A*. 2005; **102**(43): 15545-50.
39. Oughtred, R, Stark, C, Breitkreutz, BJ, *et al.* The BioGRID interaction database: 2019 update. *Nucleic Acids Res*. 2019; **47**(D1): D529-D41.
40. Kotlyar, M, Pastrello, C, Malik, Z, *et al.* IID 2018 update: context-specific physical protein-protein interactions in human, model organisms and domesticated species. *Nucleic Acids Res*. 2019; **47**(D1): D581-D9.
41. Li, T, Wernersson, R, Hansen, RB, *et al.* A scored human protein-protein interaction network to catalyze genomic interpretation. *Nat Methods*. 2017; **14**(1): 61-4.

42. Calderone, A, Castagnoli, L, Cesareni, G. mentha: a resource for browsing integrated protein-interaction networks. *Nat Methods*. 2013; **10**(8): 690-1.
43. Das, J, Yu, H. HINT: High-quality protein interactomes and their applications in understanding human disease. *BMC Syst Biol*. 2012; **6**: 92.
44. Razick, S, Magklaras, G, Donaldson, IM. iRefIndex: a consolidated protein interaction database with provenance. *BMC Bioinformatics*. 2008; **9**: 405.
45. Cowley, MJ, Pinese, M, Kassahn, KS, *et al.* PINA v2.0: mining interactome modules. *Nucleic Acids Res*. 2012; **40**(Database issue): D862-5.
46. Shannon, P, Markiel, A, Ozier, O, *et al.* Cytoscape: a software environment for integrated models of biomolecular interaction networks. *Genome Res*. 2003; **13**(11): 2498-504.
47. Thorne, LG, Bouhaddou, M, Reuschl, AK, *et al.* Evolution of enhanced innate immune evasion by the SARS-CoV-2 B.1.1.7 UK variant. *bioRxiv : the preprint server for biology*. 2021.

524 **Supplementary Figure**

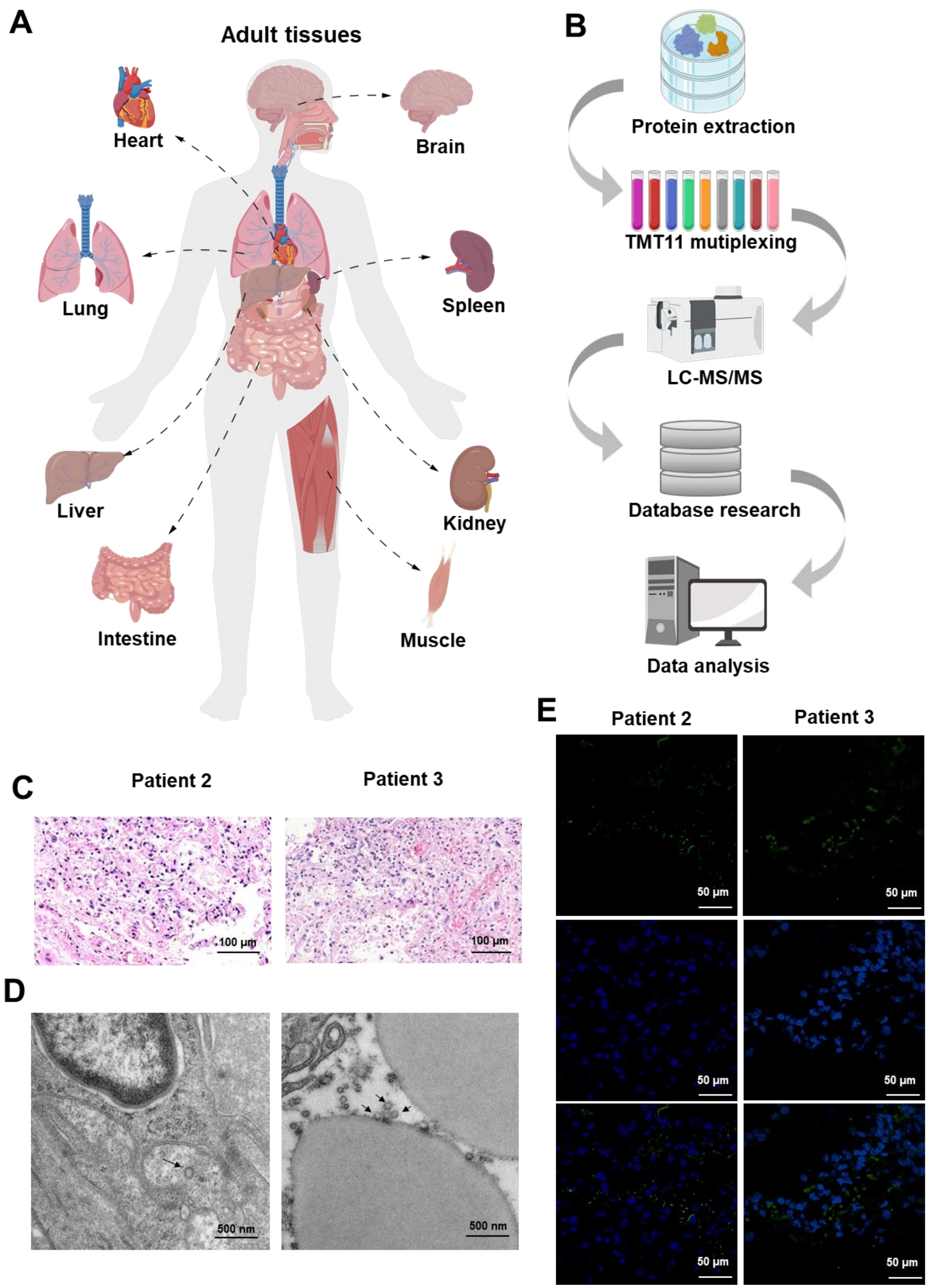

525

Figure S1. Study design and patients. A, Overview of postmortem tissue samples that were analyzed to generate a draft map of COVID-19 patient's proteome are shown. **B,** The workflow of tissue samples preparation. **C,** Pathological changes of lung tissue in two patients with COVID-19. The tissues were fixed with paraformaldehyde and stained with the hematoxylin an eosin (HE). **D,** SARS-CoV-2-like particles (black arrowed) observed by electron microscopy in lung tissues (left, original magnification 9600×; right, original magnification 7800×). **E,** SARS-CoV-2 nucleocapsid (NP) protein (green) and DAPI (blue) detected by immunofluorescence staining.

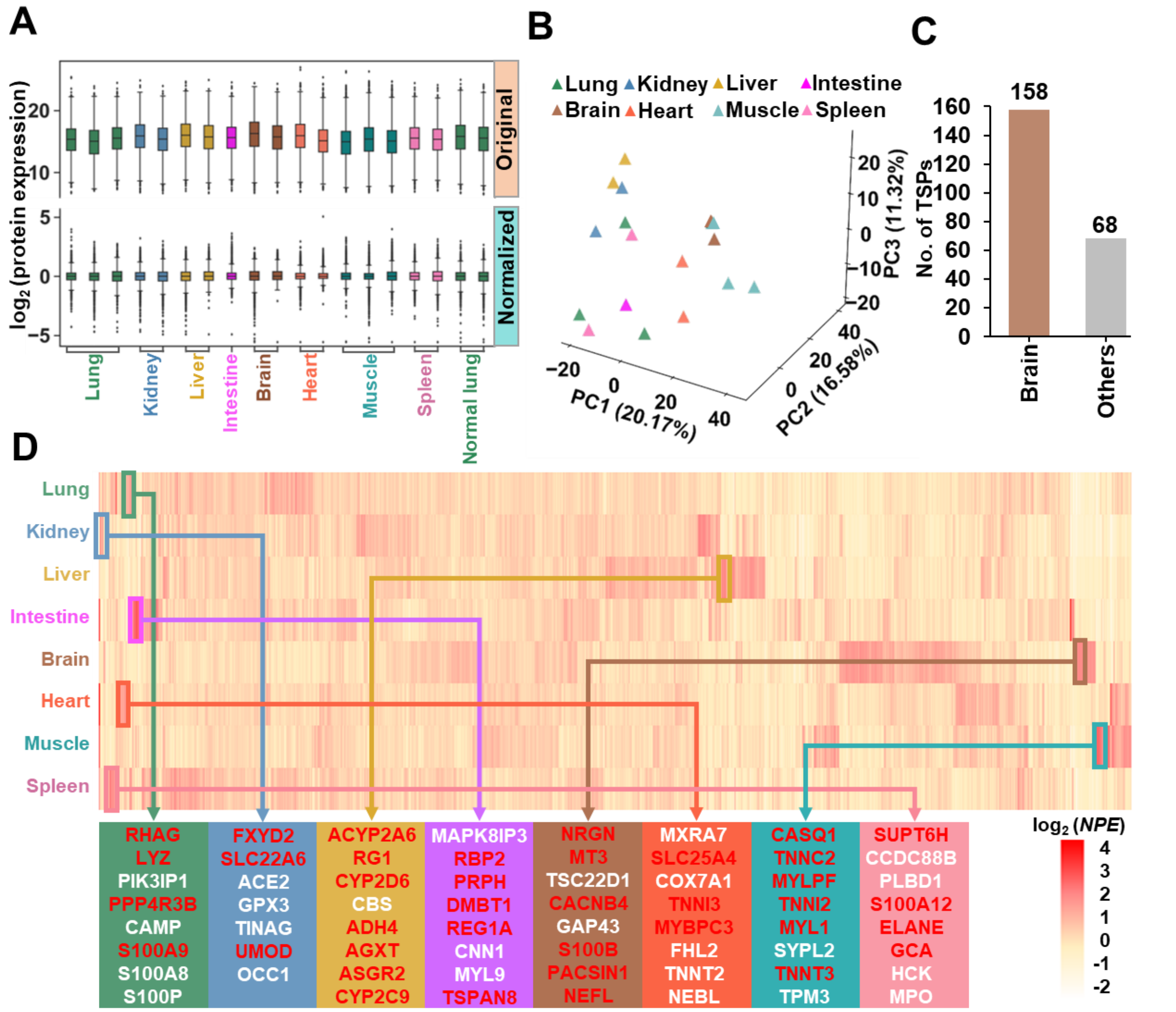

Figure S2. Proteomic profiling of eight types of COVID-19 postmortem tissues. A, Normalization of the proteomic data using the *z*-score plus min-max and median centering methods. **B,** PCA analysis of the proteomic data with *NPE* values. **C,** An entropy-based prediction of potential TSPs (entropy < 2.5). **D,** A heatmap of protein expressions in the eight types of postmortem tissues, after a hierarchical clustering. Selected proteins in boxes include well-characterized (red) and potential (white) TSPs.

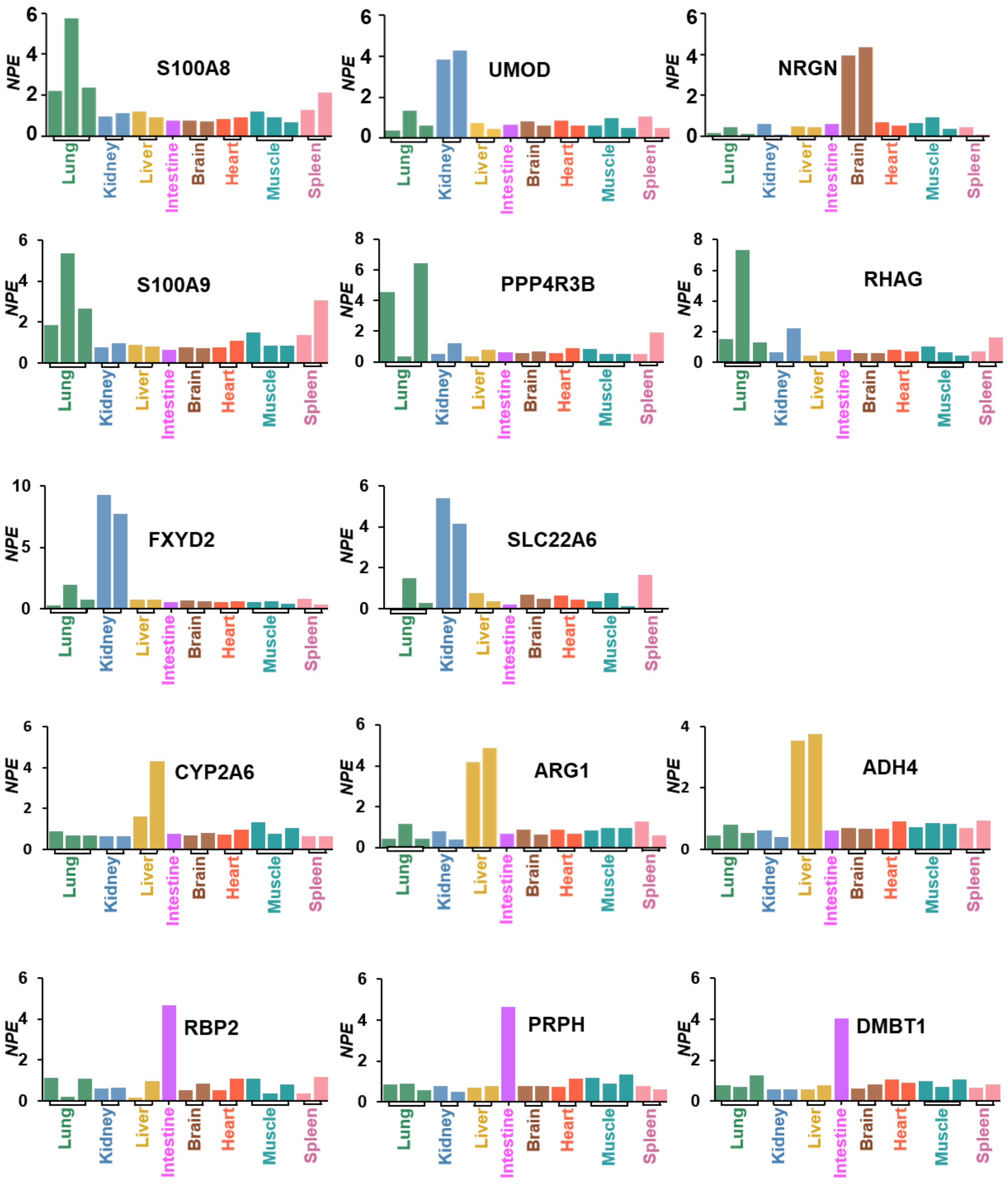

Figure S3. Normalized expression levels of the selected potentially tissue-specific proteins (TSPs) in tissues of lung, kidney, liver and intestine. The potentially TSPs were identified by the entropy-based method (Entropy score $H < 2.5$). For tissue types with multiple samples, the mean *NPE* values was calculated for each protein.

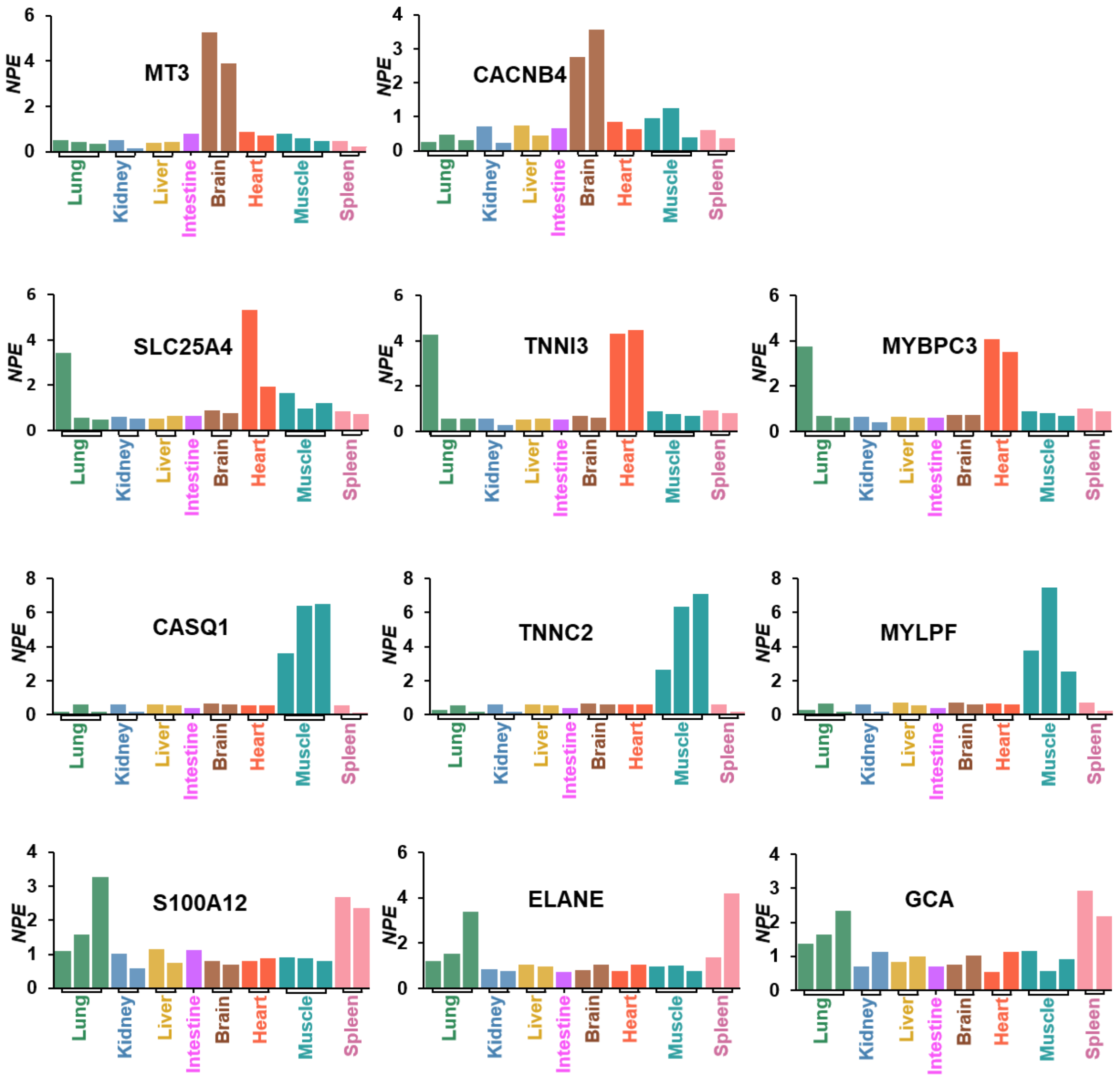

Figure S4. Normalized expression levels of the selected TSPs in tissues of brain, heart, muscle and spleen. TSPs were identified by the entropy-based method (Entropy score $H < 2.5$). For tissue types with multiple samples, the mean *NPE* values was calculated for each protein.

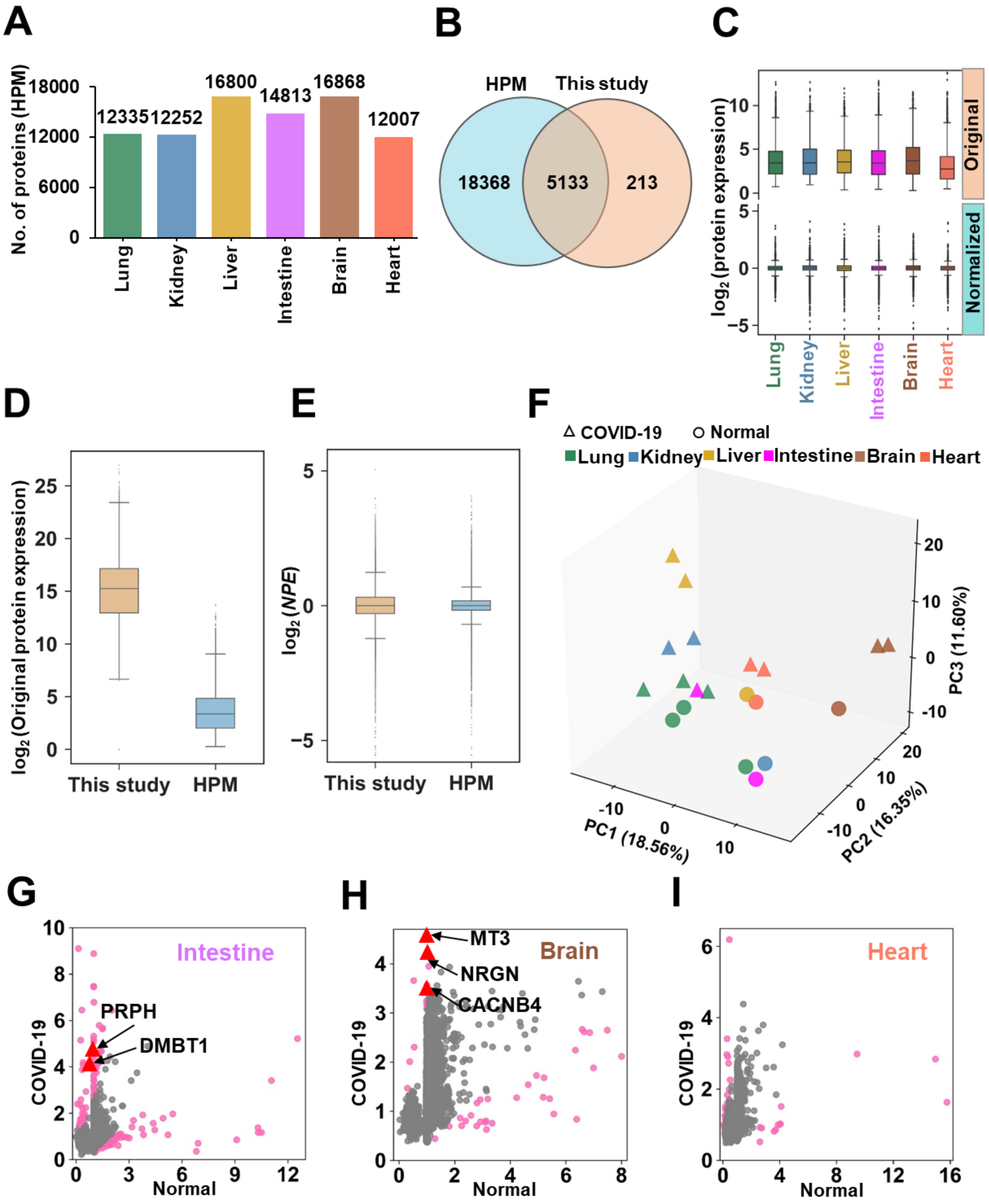

Figure S5. A comparison of the proteomic data in postmortem tissues against normal tissues in HPM. A, The distribution of numbers of quantified proteins for six tissues obtained from HPM. **B,** The overlap of quantified proteins in HPM and this study. **C,** The *z*-score plus min-max and

median centering normalizations of the HPM proteomic data. **D,** The distribution of protein expressions of our and HPM data sets before normalization. **E,** The distribution of NPE values of our and HPM data sets after normalization. **F,** The PCA separation of COVID-19 and normal tissues, irrespective of the data source. **G-I,** MAP-based identification of potential DEPs in (G) intestine, (H) brain and (I) heart of postmortem tissues.

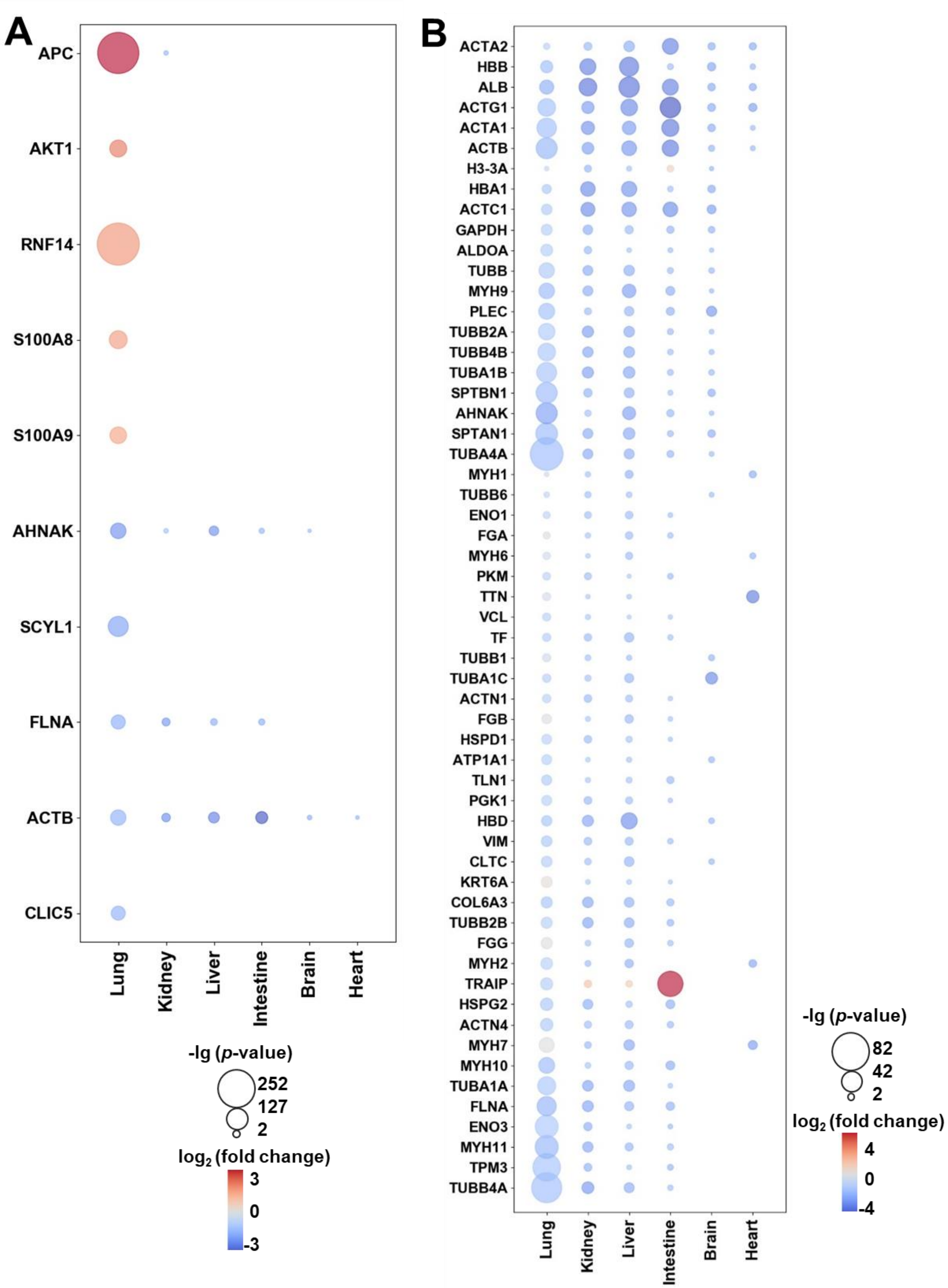
A
APC
AKT1
RNF14
S100A8
S100A9
AHNAK
SCYL1
FLNA
ACTB
CLIC5
Lung
Kidney
Liver
Intestine
Brain
Heart
-lg (p-value)
252
127
2
log2 (fold change)
3
0
-3
B
ACTA2
HBB
ALB
ACTG1
ACTA1
ACTB
H3-3A
HBA1
ACTC1
GAPDH
ALDOA
TUBB
MYH9
PLEC
TUBB2A
TUBB4B
TUBA1B
SPTBN1
AHNAK
SPTAN1
TUBA4A
MYH1
TUBB6
ENO1
FGA
MYH6
PKM
TTN
VCL
TF
TUBB1
TUBA1C
ACTN1
FGB
HSPD1
ATP1A1
TLN1
PGK1
HBD
VIM
CLTC
KRT6A
COL6A3
TUBB2B
FGG
MYH2
TRAIP
HSPG2
ACTN4
MYH7
MYH10
TUBA1A
FLNA
ENO3
MYH11
TPM3
TUBB4A
Lung
Kidney
Liver
Intestine
Brain
Heart
-lg (p-value)
82
42
2
log2 (fold change)
4
0
-4

Figure S6. Normalized expression levels of DEPs. **A,** Normalized expression levels of the most changed DEPs in lungs and other tissues. **B,** Normalized expression levels of 57 DEPs shared by more than 4 tissues. DEPs were identified by MAP (Adjusted *p*-value < 0.05). For tissue types with multiple samples, the mean *NPE* values was calculated for each protein.

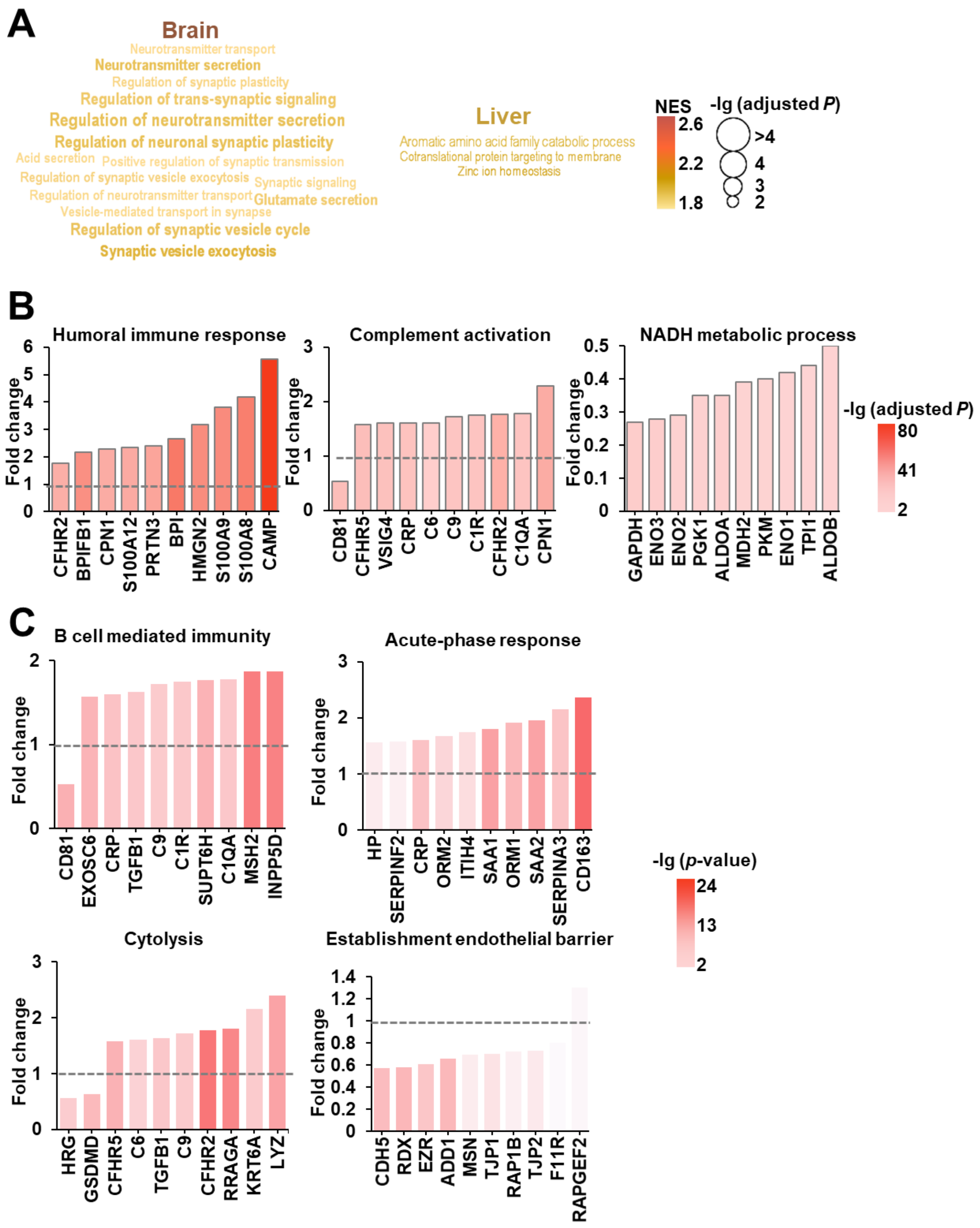

Figure S7. Additional analyses of DEPs. **A,** Visualization of up-regulated processes in postmortem brain and liver, using the word cloud illustrator WocEA. **B,** Top 10 mostly changed DEPs in three

differentially regulated processes. **C,** Normalized expression levels of lung DEPs in the differentially regulated processes. GSEA analysis of lung DEPs in additional four processes. The mean *NPE* values was calculated for each protein.

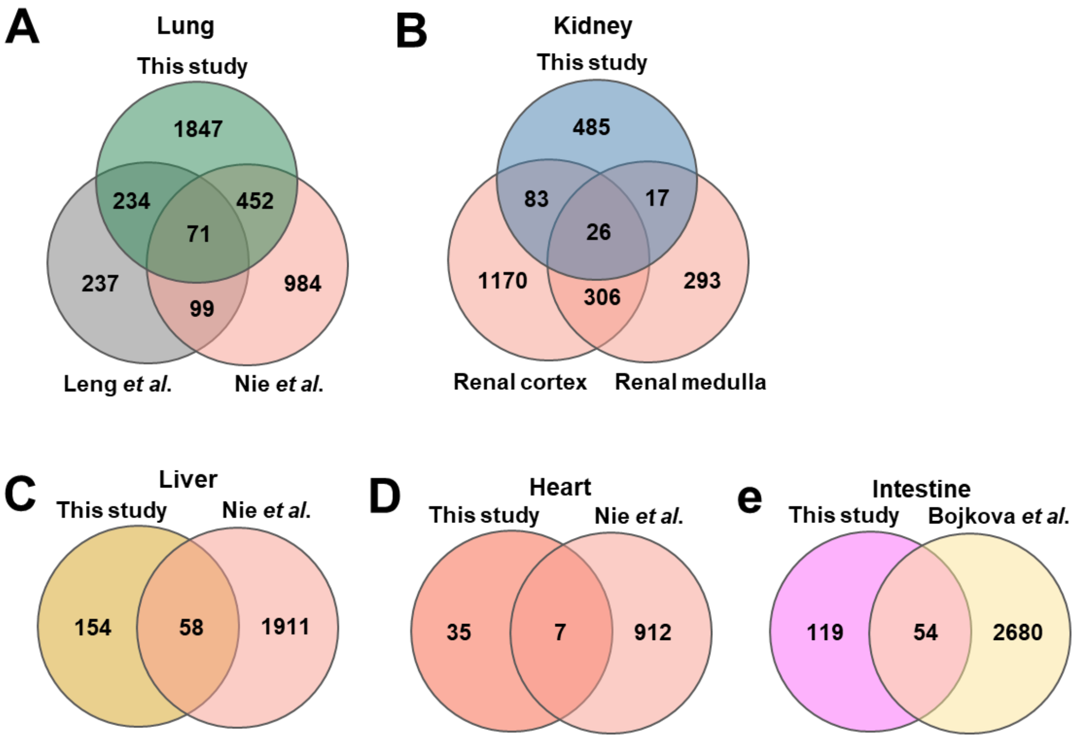

Figure S8. Comparison of the DEPs identified in this study to other published proteomic studies [24, 28, 31], including in **A,** lung, **B,** kidney, **C,** liver, **D,** heart, and **E,** intestine. More details were shown in Table S9.

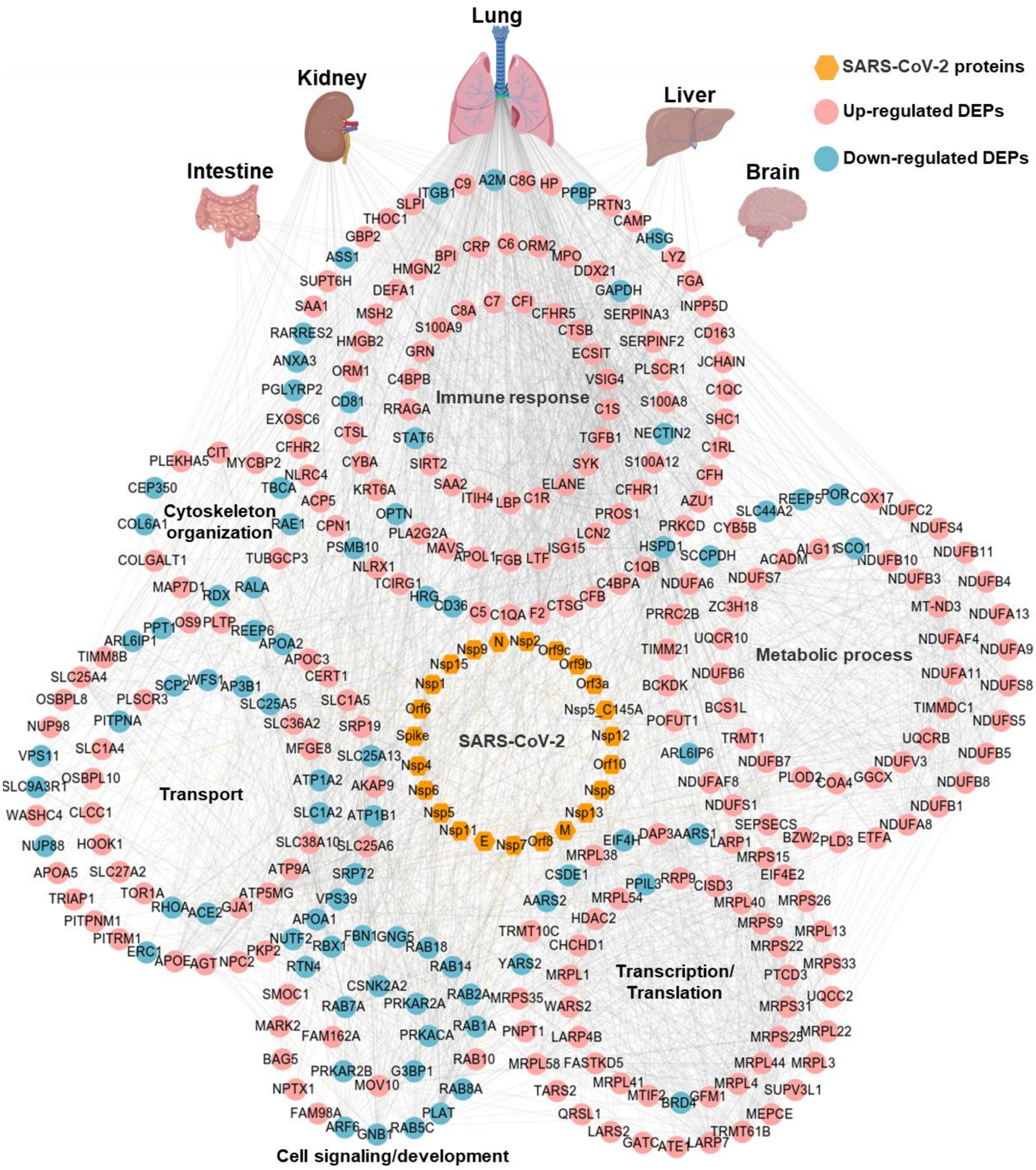

Figure S9. A virus-host protein interaction network. In the network, the 308 up- (pink) and down-regulated (cyan) DEPs in postmortem lung tissues were classified into 6 groups based on their major functions. The PPIs between the 23 SARS-CoV-2 proteins (orange) were shown in yellow links, whereas PPIs between host proteins were shown in grey links.

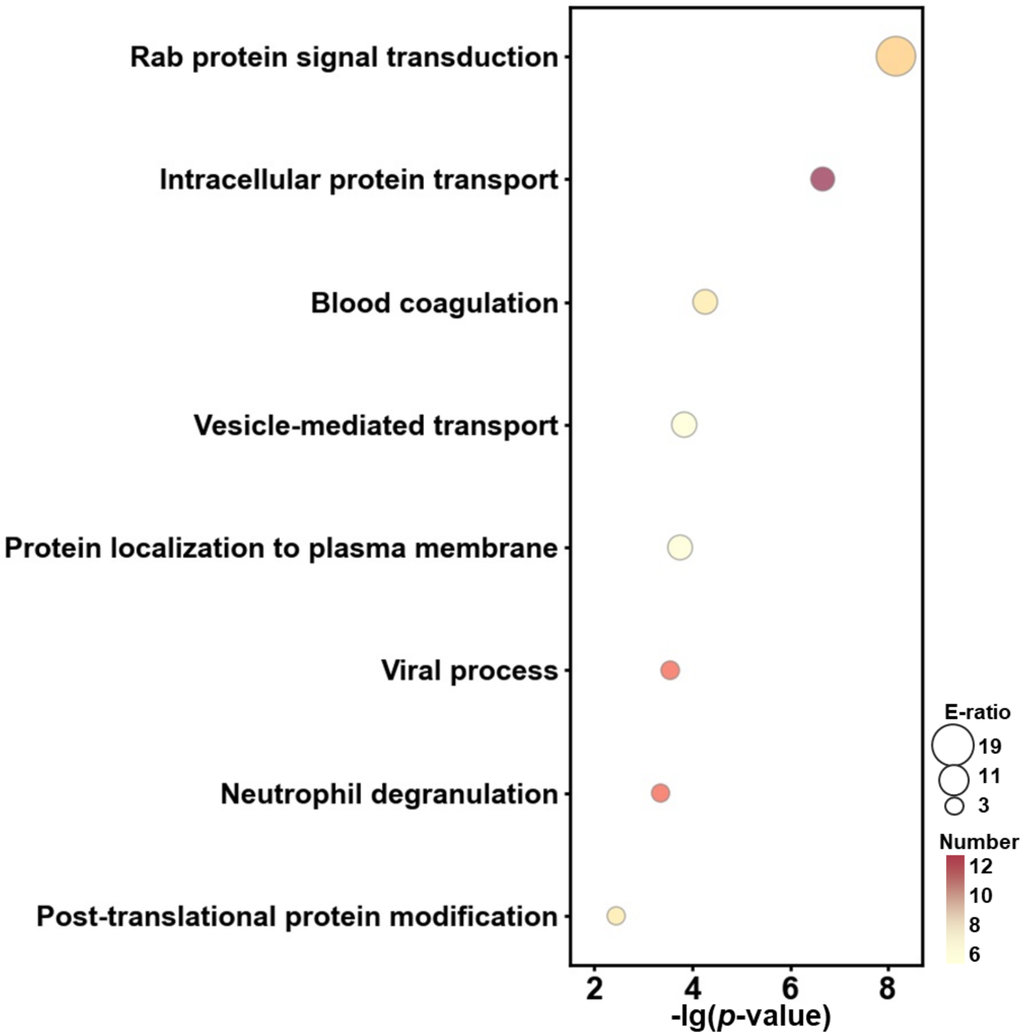

Fig. S10. GO analysis of 110 SARS-CoV-2 interacting DEPs in lung tissues. GO enrichment analysis of SARS-CoV-2 interacting DEPs. Two-sided hypergeometric test, $m > 5$, P value < 0.01.
